# Identification of Preisach Model Parameters Based on an Improved Particle Swarm Optimization Method for Piezoelectric Actuators in Micro-Manufacturing Stages

**DOI:** 10.3390/mi13050698

**Published:** 2022-04-29

**Authors:** Lei Yang, Bingxiao Ding, Wenhu Liao, Yangmin Li

**Affiliations:** 1 School of Information Science and Engineering, Jishou University, Jishou 416000, China; leiyyang@hotmail.com (L.Y.); whliao2007@aliyun.com (W.L.); 2 College of Physics and Electromechanical Engineering, Jishou University, Jishou 416000, China; 3 Department of Industrial and Systems Engineering, The Hong Kong Polytechnic University, Hong Kong 999077, China

**Keywords:** Preisach hysteresis, piezoelectric materials, improved particle swarm optimization

## Abstract

The Preisach model is a typical scalar mathematical model used to describe the hysteresis phenomena, and it attracts considerable attention. However, parameter identification for the Preisach model remains a challenging issue. In this paper, an improved particle swarm optimization (IPSO) method is proposed to identify Preisach model parameters. Firstly, the Preisach model is established by introducing a Gaussian−Gaussian distribution function to replace density function. Secondly, the IPSO algorithm is adopted to Fimplement the parameter identification. Finally, the model parameter identification results are compared with the hysteresis loop of the piezoelectric actuator. Compared with the traditional Particle Swarm Optimization (PSO) algorithm, the IPSO algorithm demonstrates faster convergence, less calculation time and higher calculation accuracy. This proposed method provides an efficient approach to model and identify the Preisach hysteresis of piezoelectric actuators.

## 1. Introduction

Piezoelectric actuators (PEAs) are widely utilized in the demanding field of high-precision motion for their advantages of high resolution, large driving force, high stiffness, small volume and high reliability [1,2]. PEAs use inverse piezoelectric characteristics to achieve continuous output motion [3]. However, the inherent nonlinearity hysteresis, multivalued mapping and rate-independent phenomenon lead to poor accuracy, and they easily generate oscillation, which greatly affects the positioning precision of PEAs [4].

**Figure 1:**
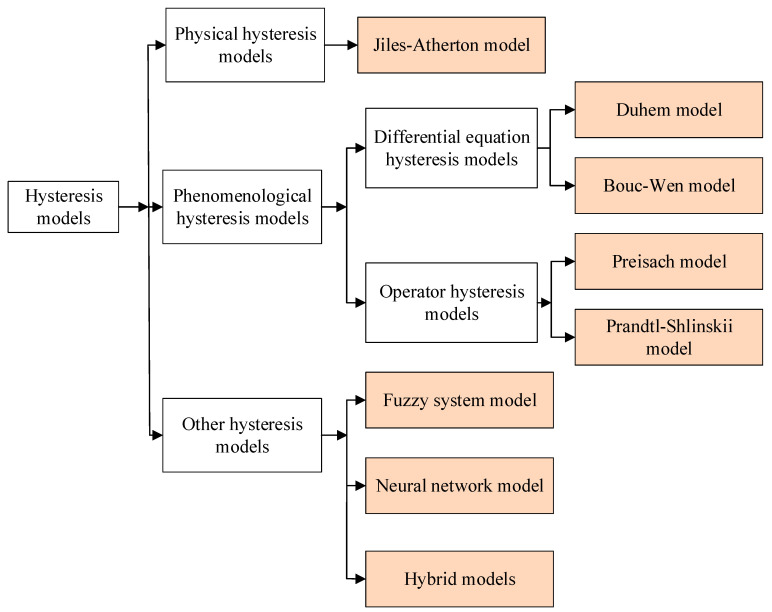
The classification diagram of hysteresis models.

In recent years, smart material-based actuators have attracted extensive attention and many mathematical models have been proposed to describe their hysteresis characteristics. Generally speaking, these models can be classified as physics-based hysteresis models [5,6] and phenomenology-based hysteresis models [7,8,9]. Also, there are other hysteresis models, including the neural network model, the fuzzy system model and hybrid models [10,11,12]. The detailed classification of hysteresis models is shown in Figure 1.

Physics-based hysteresis models are based on the first nature principle, the minimum free energy and the stress−strain relationship and study the electric dipole, electric domains and their movement laws from the microscopic mechanism of the interaction between the nucleus and the electron. The most typical physical model is the hysteresis model of ferromagnets (Jiles−Atherton model), which was proposed by Jiles and Atherton in 1986 [13]. For phenomenological models, the behavior of the material is described mathematically by generating curves and following predefined rules for the material properties [14]. The differential equation model and the operator hysteresis model are two typical phenomenological models. The differential equation models employ nonlinear differential equations to describe the hysteresis [15]. Examples of differential equation models include the Bouc−Wen model [16] and the Duhem model [17]. The operator hysteresis model employs a weighted superposition of nonlinear operators to describe the hysteresis [18]. Examples of operator hysteresis models include the Preisach model [19] and the Prandtl−Ishlinskii model [20]. The Preisach model is a typical scalar model to describe hysteresis phenomena. It has attracted considerable attention because of its ability to describe the hysteresis loop accurately. However, the process to obtain the density function of the Preisach model is difficult. To address this issue, a Gaussian−Gaussian distribution function approximating the Preisach model density function was adopted to model the hysteresis of PEAs.

In addition, the parameter identification for the Preisach model is a challenging problem. Some parametric identification approaches to identify these parameters have been reported, such as the genetic algorithm (GA), differential evolution (DE), the least squares method (LSM) and the PSO algorithm [21,22,23]. For instance, Hergli et al. used the GA to identify parameters of the Preisach model [24]. Nevertheless, the main drawbacks of the GA are the high computational burden and the slow convergent rate near the global optimum. The GA can obtain a value near the global minimum but cannot guarantee attainment of the global minimum. DE also demonstrates the characteristics of a slow convergent rate when used to obtain the global optimum. On the other hand, the PSO algorithm has been proven to be a robust intelligent optimization algorithm, which can be easily understood and implemented. However, the PSO algorithm is easy to fall into the local optimum when dealing with complex nonlinear problems with high dimensionality, which leads to large errors in optimization results. For these reasons, the Preisach hysteresis model parameters are identified by the IPSO algorithm in this paper.

This paper describes an approach to identify Preisach model parameters using the IPSO method. The proposed identification method has achieved significant improvements in both accuracy and computational time when compared with the PSO approach. To demonstrate the efficiency of this identification method, simulation studies were conducted via MATLAB software. The rest of this paper is organized as follows: in Section 2, the classical Preisach model and the key issues associated with the implementation of the Preisach model are introduced; then, the Preisach model based on the Gaussian−Gaussian distribution function is developed in Section 2; the PSO algorithm is improved in Section 3; to validate the effectiveness of the proposed method, experimental studies are added to obtain optimal results in Section 4; finally, this work is summarized in Section 5.

## 2. Preisach Model

### 2.1. Preisach Hysteresis Operator

**Figure 2:**
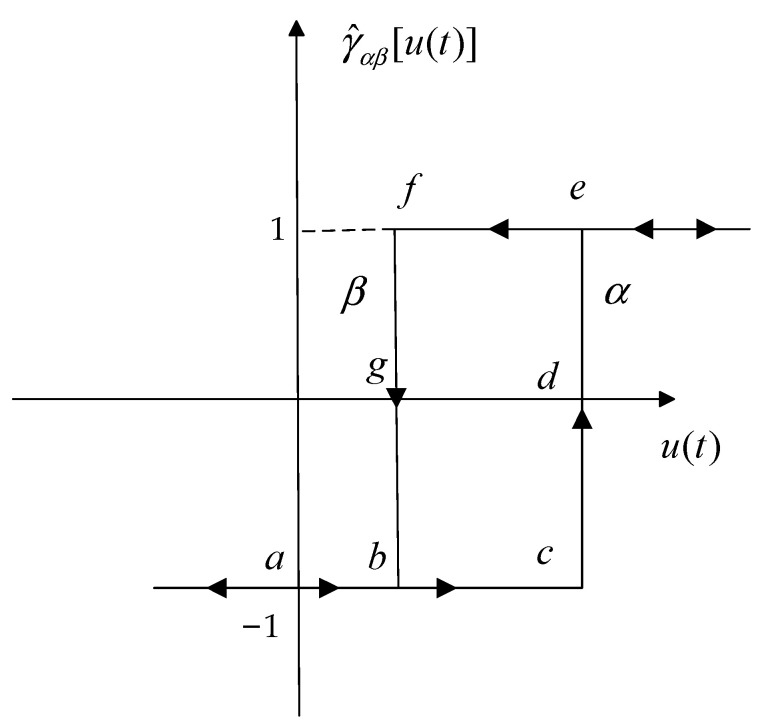
Elementary hysteresis operator.

The Preisach operator is developed by the delayed relay operator ${\hat{\gamma }}_{\alpha \beta }[u(t)]$. The illustration of ${\hat{\gamma }}_{\alpha \beta }[u(t)]$ is depicted in Figure 2, and the ${\hat{\gamma }}_{\alpha \beta }[u(t)]$ can be written as:

(1) $${\hat{\gamma }}_{\alpha \beta }[u(t)]=\left\{ \begin{array}{cc} 1 & u(t)>\alpha \\ -1 & u(t)<\beta \\ {\hat{\gamma }}_{\alpha \beta }[u(t-1)] & \beta <u(t)<\alpha \end{array}\right.$$

where *t* denotes the time, *u*(*t*) represents the input voltage, α and β are the upper and lower thresholds, respectively, and they are defined from the previous output ${\hat{\gamma }}_{\alpha \beta }[u(t-1)]=\xi$, where ξ∈{+1,−1} is the state of the relay and ${\hat{\gamma }}_{\alpha \beta }$ is the output of the relay corresponding to the (α,β) pair.

**Figure 3:**
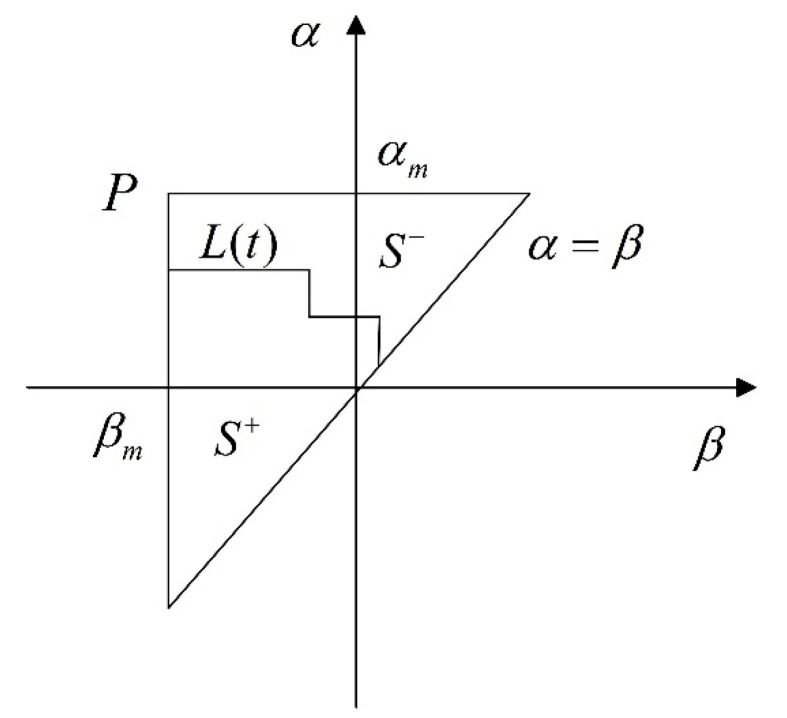
Preisach plane.

In addition, the output of the Preisach model is calculated as a weighted superposition of delayed relays. The Preisach model can be expressed as follows:

(2) $$f(t)=\iint_{\alpha \geq \beta }\mu (\alpha ,\beta ){\hat{\gamma }}_{\alpha \beta }[u(t)]d\alpha d\beta$$

where f(t) is the output displacement and μ(α,β) denotes the density function. The density function μ(α,β) is a non-negative weight function, representing the weights of each hysteron in the Preisach plane $P=\{(\alpha ,\beta ):\alpha \geq \beta ,\alpha \leq \alpha_{m},\beta \geq \beta_{m}\}$, where $\alpha_{m}$ and $\beta_{m}$ refer to the highest and lowest values of α and β, respectively. The model output can be calculated using the double integral in the plane region *P* of the Preisach model, as shown in Figure 3.

In Figure 3, *L*(*t*) is the step line, which separates the Preisach plane into positive and negative areas, reflecting the magnetization history. $S^{+}$ corresponds to relays with output values of +1 and $S^{-}$ corresponds to output values of −1.

Based on the regions $S^{+}$ and $S^{-}$, the output in (1) can be rewritten as:

(3) $$f(t)=\iint_{s^{+}}\mu (\alpha ,\beta )d\alpha d\beta -\iint_{s^{-}}\mu (\alpha ,\beta )d\alpha d\beta$$

Referring to Equation (3), the determination of the density function is the key to obtain the value of f(t). Therefore, the parameter identification of the Preisach model synonymous with the parameter identification of the density function. However, this process requires a large amount of experimental data. In addition, the first-order reversal loop of the hysteresis loop needs to be measured, which requires a large workload and results in low accuracy. It has become a challenging task to identify Preisach model parameters.

### 2.2. Implementation of the Preisach Model

In order to overcome the difficulties associated with the determination of the density function of the Preisach model, it is necessary to reduce dependence on the first-order reversal loop data. Moreover, reducing the complexity and computational workload of the double integration is also important for the attainment of the Preisach model. For this purpose, the Gaussian−Gaussian distribution function is introduced [25], which replaces the density function for two Gaussian probability distributions. The expression for Gaussian−Gaussian distribution function is depicted as follows:

(4) $$\mu^{\prime \prime }(\alpha ,\beta )=\frac{1}{2\pi \sigma_{c}\sigma_{u}H_{0}{}^{2}}\frac{1}{erf(\sigma_{c}\sqrt{2})+1}\exp\left[-\frac{{\left(\frac{\left(\alpha -\beta \right)}{2}-H_{0}\right)}^{2}}{2\sigma_{c}{}^{2}H_{0}{}^{2}}-\frac{{\left(\frac{\left(\alpha +\beta \right)}{2}\right)}^{2}}{2\sigma_{u}{}^{2}H_{0}{}^{2}}\right]$$

where $\sigma_{c}$, $\sigma_{u}$ is the standard deviation on each respective diagonal, $H_{0}$ is the maximum position and erf is the error function, which is defined as:

(5) $$erf(x)=\frac{2}{\pi }\int_{0}^{x}\exp(-u^{2})du$$

when $\sigma_{c}=\sigma_{u}=H_{0}=1$, the following expression is obtained:

(6) $$\mu^{\prime \prime }(\alpha ,\beta )=\frac{1}{2\pi }\frac{1}{erf(\sqrt{2})+1}\exp\left[-\frac{{\left(\frac{\left(\alpha -\beta \right)}{2}-1\right)}^{2}-{\left(\frac{\left(\alpha +\beta \right)}{2}\right)}^{2}}{2}\right]$$

**Figure 4:**
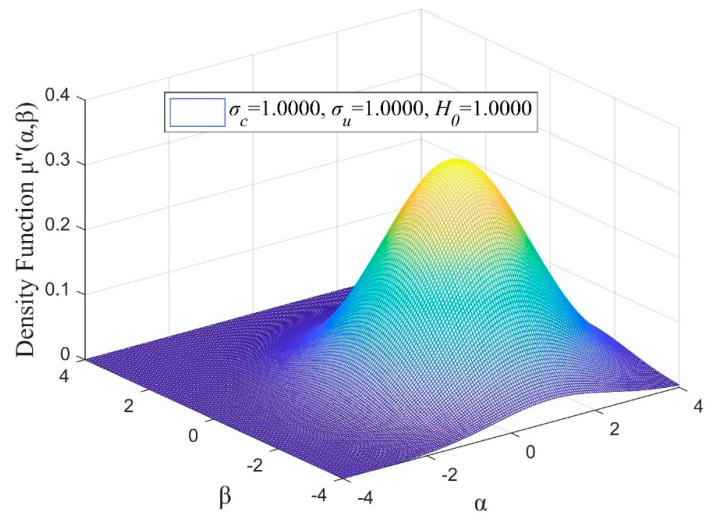
Plot of the *μ*″(*α*, *β*) with *σ_c_* = 1, *σ_u_* = 1 and *H*_0_ = 1.

Figure 4 depicts the distribution of the density function $\mu^{\prime \prime }(\alpha ,\beta )$.

Thus, the expression of the Preisach model can be determined as:

(7) $$f(t)=\iint_{\alpha \geq \beta }\mu^{\prime \prime }(\alpha ,\beta ){\hat{\gamma }}_{\alpha \beta }[u(t)]d\alpha d\beta$$

Referring to Equation (7), the parameters that need to be identified in the Preisach model based on the Gaussian−Gaussian distribution function are $\sigma_{c}$, $\sigma_{u}$ and $H_{0}$. Compared with the classical Preisach model, this model has a more concise expression and fewer parameters. Thus, it not only improves the computational efficiency of the Preisach model, but also reduces the difficulty of parameter identification.

## 3. Parameter Identification Based on the IPSO Algorithm

### 3.1. Determination of the Fitness Function

The key problem of Preisach model parameter identification is the selection of the fitness function, which is used to get a minimum value with a root-mean-square between the actual system and the Preisach model. In this study, the objective function is chosen as:

(8) $$\eta =\sqrt{\frac{1}{N}\sum_{k=1}^{N}{\left(Y_{m}(\sigma_{c},\sigma_{u},H_{0})-Y\right)}^{2}}$$

where η denotes the root-mean-square error and Y and $Y_{m}$ denotes the output experimental value and the analytical value, respectively.

### 3.2. Improvement of the PSO Algorithm

**Figure 5:**
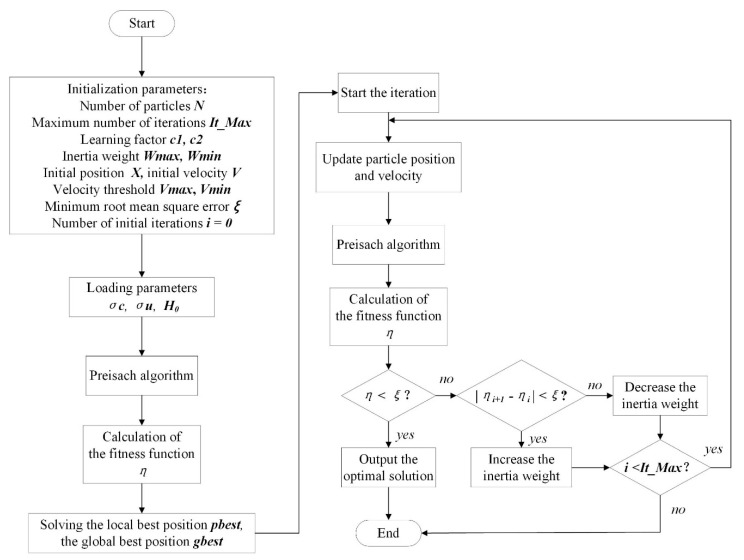
IPSO algorithm identification process flowchart.

The PSO algorithm has the advantages of simple and easy implementation, fast convergence and few parameters to be adjusted. However, the PSO algorithm is easy to fall into the local optimum when dealing with complex nonlinear problems with high dimensionality, which leads to large errors in the optimization results. To address the limitations of the PSO algorithm, several improvements are made to the PSO algorithm in this paper, including setting the minimum error value $\xi =80e^{80\eta -8}$. Additionally, during the iteration process, the inertia weight decreases amid the difference of the fitness function values between two consecutive particles $|\eta_{i+1}-\eta_{i}|>\xi$. Otherwise, we can increase the inertia weight. On this basis, the adaptive effect on inertia weights is achieved, and the success rate and the convergence speed of the search are improved. The parameter identification process of the IPSO algorithm is shown in Figure 5.

During the IPSO algorithm identification process, the evaluation is carried out for an initial population of 10 individuals and 100 iterations. The learning factors *c*1 and *c*2 are both equal to 1.5. The lower and upper thresholds of the Preisach model parameters {$\sigma_{c}$, $\sigma_{u}$, $H_{0}$} to be identified are {0, 0, 1} and {1, 3, 100}, respectively. Once the iteration is started, the Preisach model calculates the displacement, which is then evaluated using the fitness function ς. The fitting results between the measured and simulated values are provided for each iteration process and compared with the minimum error ($\xi =80e^{80\eta -8}$).

## 4. Experimental Simulation

### 4.1. Hysteresis Loop for PEAs

**Figure 6:**
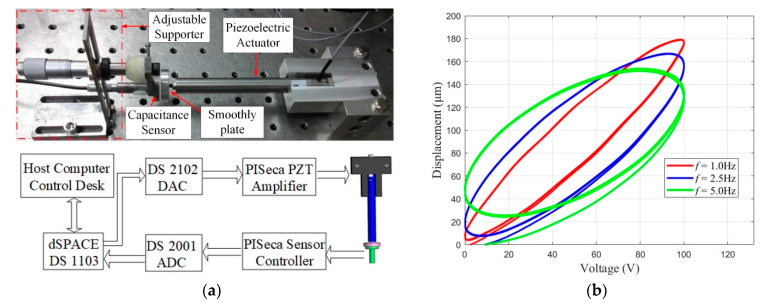
(**a**) The scheme of the experimental setup and hardware connection. (**b**) The static hysteresis loop of piezoelectric ceramic materials at different frequencies.

In order to implement the parameter identification of the Preisach model, the static hysteresis loop data needs to be measured. A preloaded PEA P-840.60 from Physik Instruments is adopted, with a nominal travel range of 90 μm and an input voltage of 0–100 V [26]. The PEA is driven by a voltage amplifier device, and the amplification ratio is 10. The output displacement of the PEA is measured by the capacitance sensor (D-510 from Physik Instrument) with characteristics such as a sub-nanometer resolution and linearity better than 0.1%, a nominal measuring range of 100 μm and a bandwidth of up to 10 kHz [27]. The excitation signals are generated by dSPACE, and the setup of the instruments are shown in Figure 6a. The displacement data in the frequency of 1.0 Hz, 2.5 Hz and 5.0 Hz were recorded, as shown in Figure 6b.

### 4.2. Preisach Model Hysteresis Loop

**Figure 7:**
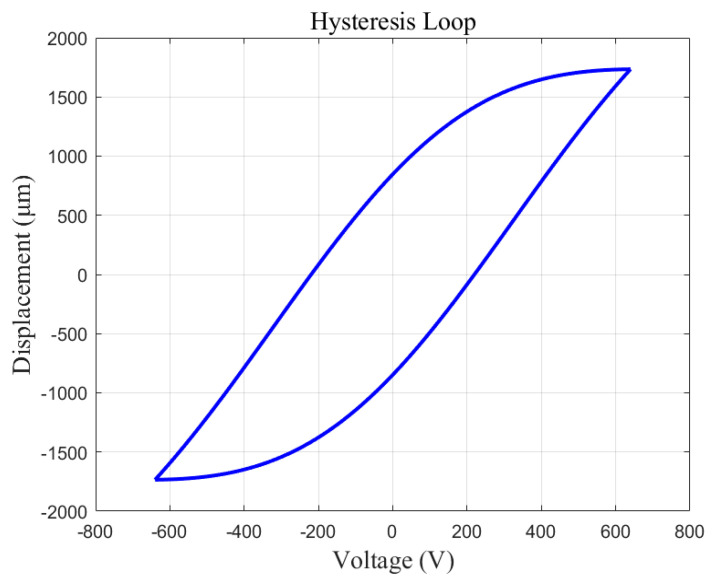
Preisach hysteresis loop.

A Preisach model based on the Gaussian−Gaussian distribution function is developed in this paper. Assuming that the input voltage interval of the model is [−650, 650], the obtained hysteresis loop is shown in Figure 7.

**Figure 8:**
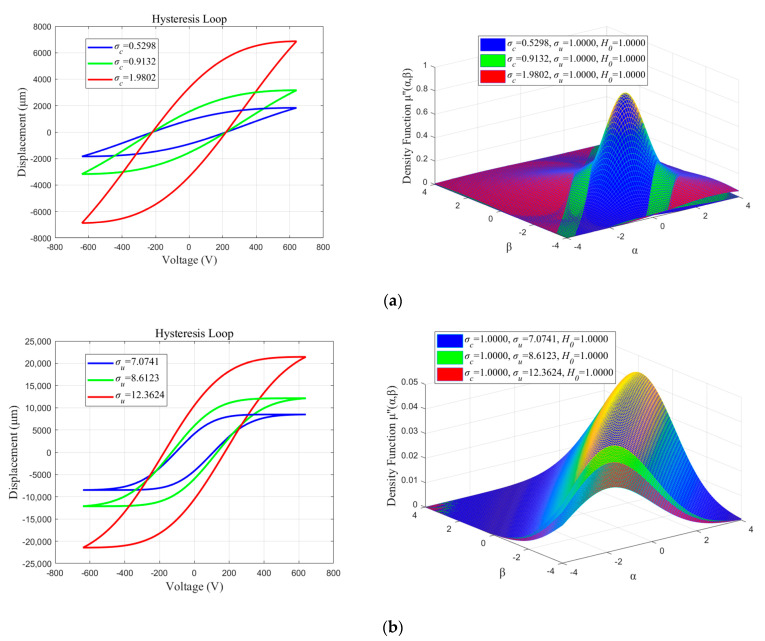

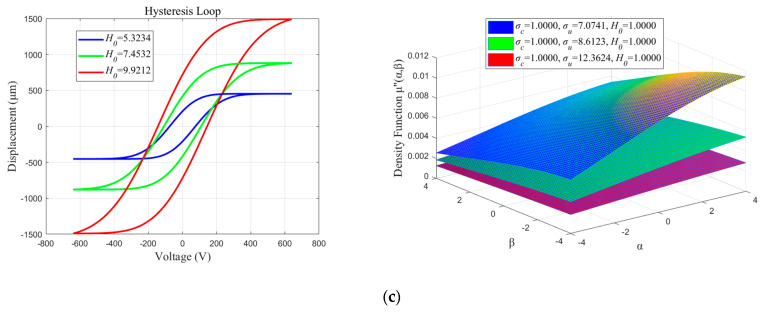
The parameters’ effect on hysteresis loop: (**a**) Effects of parameter *σ_c_*; (**b**) Effects of parameter *σ_u_*; (**c**) Effects of parameter *H*_0_.

To analyze the effects of parameters $\sigma_{c}$, $\sigma_{u}$ and $H_{0}$ on the model, two parameters remain constant while the rest of the parameters are changed. Referring to Figure 8, the slope and width of the hysteresis loop increases with increasing values of $\sigma_{c}$, and the upper and lower thresholds of the output values changes less. As $\sigma_{u}$ increases, the slope of the hysteresis loop essentially remains constant; however, the width increases, which has a greater effect on the upper and lower thresholds of the output values. As $H_{0}$ increases, the slope and width of the hysteresis loop essentially remain constant, while the upper and lower thresholds of the output values change significantly.

### 4.3. Parameter Optimization of the Preisach Model

**Figure 9:**
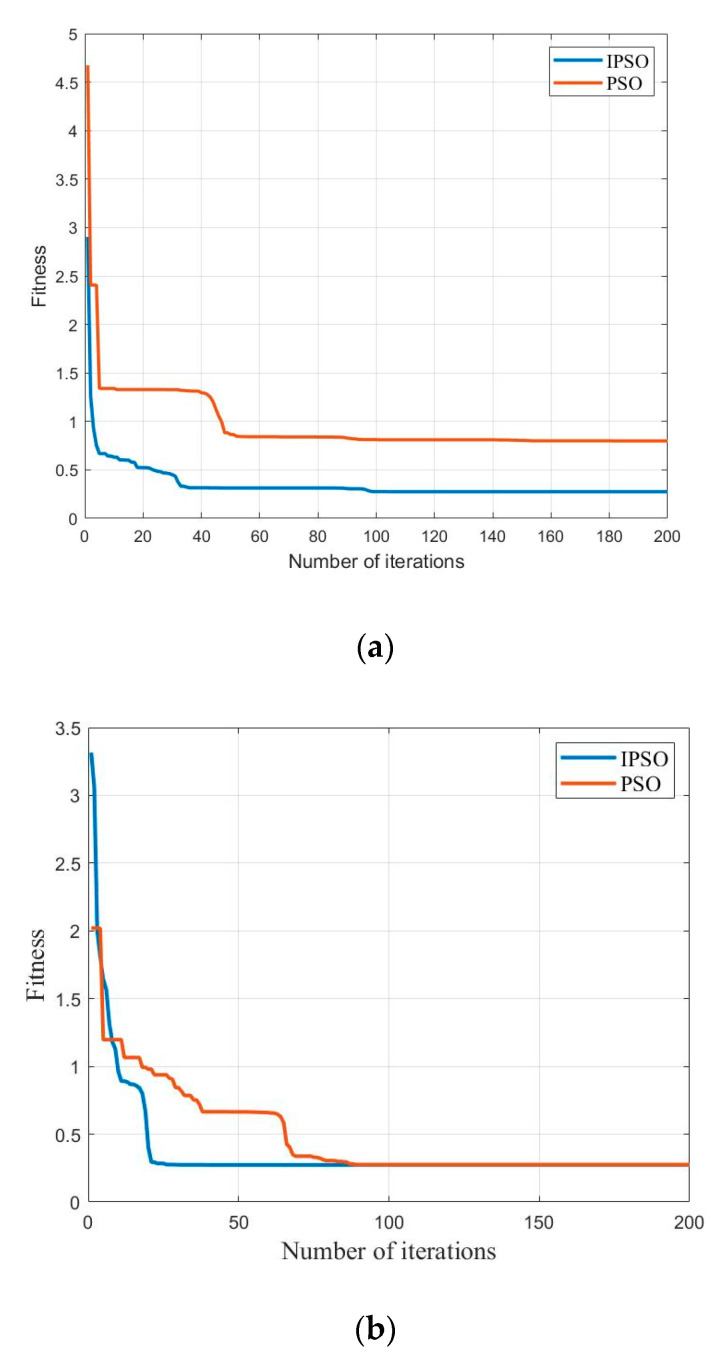
Comparison of the iterative process of IPSO and PSO algorithms; (**a**) iteration process for ascending curve; (**b**) iteration process for descending curve.

According to the IPSO identification process shown in Figure 5, 500 samples were selected from experimental data using *f* = 5.0 Hz as an input. To further verify the accuracy of the model, the upper and lower bound curves of the Preisach model are identified. The obtained results of the model parameters are listed in Table 1. The iteration process of the algorithm is shown in Figure 9. Further, it should be noted that the control signal of the piezoelectric actuator 0–100 V and the actual displacement 0–100 μm are normalized to the range of −1–0 in the identification process for the sake of convenience.

The comparison curves of the iteration process are depicted in Figure 9; these curves use PSO and IPSO algorithms corresponding to ascending and descending curves. Referring to this Figure, the IPSO algorithm demonstrates advantages of faster convergence speed and higher computational accuracy during the iterative process. It can meet the requirements of parameter identification for the Preisach model.

**Figure 10:**
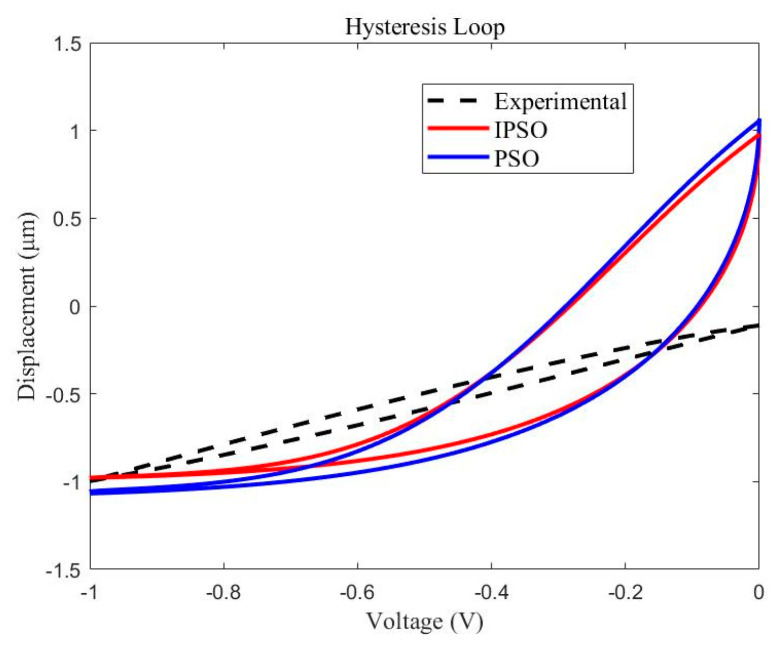
Parameter identification results and comparisons.

After substituting the identified parameter results of the IPSO and PSO into the Preisach model, the Preisach hysteresis loop can be obtained by MATLAB, as shown in Figure 10.

The comparison between the simulated values and the experimental data reveals that the parameter identification results of the IPSO are significantly better than those of the PSO algorithm. However, there is also a certain number of errors between the analytical curves and the experimental curves, which shows unsuccessful modeling. As observed in Figure 10, when the unipolar loading is applied, a very large error is produced between the model curve and the experimental curve, even though the root-mean-square error is very small. Therefore, the method that uses the Preisach model based on the Gaussian−Gaussian distribution function to model the hysteresis phenomenon may not be sufficiently accurate.

## 5. Conclusions and Future Works

A parametric identification method for the Preisach model based on the IPSO algorithm is proposed in this paper. The Gaussian−Gaussian distribution function is introduced to replace the density function. Then, the Preisach model based on the Gaussian−Gaussian distribution function is developed, and a parametric sensitivity analysis for hysteresis property is conducted. The IPSO approach is utilized to identify the parameters of this model. The comparison of results showed that the IPSO algorithm is better than the PSO algorithm in terms of accuracy and convergence time. Considering the results obtained in this paper, the use of the Preisach hysteresis modeling method based on the Gaussian−Gaussian distribution function cannot adequately describe the actual hysteresis measurements. In addition, the calculation of the ascending and descending regions of the Preisach memory curve using this method is time-consuming. However, reducing the number of measurement points of the input signal degrades the accuracy of the calculated hysteresis loops. The aim of our future work is to improve the model accuracy of the proposed method without massive computations. In addition, we will apply this model or establish a generalized model to describe smart materials hysteresis.

## Figures and Tables

**Table 1:** Identification results of upper and lower bound curve parameters of the Preisach model.

Algorithm	Parameters					
	Ascending Curve			Descending Curve		
	$\sigma_{c}1$	$\sigma_{u}1$	$H_{0}1$	$\sigma_{c}2$	$\sigma_{u}2$	$H_{0}2$
**IPSO**	0.0012	0.6500	1.7302	0.0023	0.6990	51.2568
**PSO**	0.0034	0.6783	3.9821	0.3821	0.9213	32.9821

## Data Availability

Not applicable.

## References

[1] Devasia S., Eleftheriou E., Moheimani S.O.R. (2007). A survey of control issues in nanopositioning. IEEE Trans. Control Syst. Technol..

[2] Hubbard N.B., Culpepper M.L., Howell L.L. (2006). Actuators for micropositioners and nanopositioners. Appl. Mech. Rev..

[3] Ding B., Li Y. (2018). Hysteresis Compensation and Sliding Mode Control with Perturbation Estimation for Piezoelectric Actuators. Micromachines.

[4] Alem S., Izadi I., Sheikholeslam F. (2017). Adaptive Sliding Mode Control of Hysteresis in Piezoelectric Actuator. IFAC-PapersOnLine.

[5] Miri N., Mohammadzaheri M., Chen L. (2014). An enhanced physics-based model to estimate the displacement of piezoelectric actuators. J. Intell. Mater. Syst. Struct..

[6] Miri N., Mohammadzaheri M., Chen L. (2015). An evolutionary approach to physics-based modelling of piezoelectric actuators, supported by a critical review and experimental results. Int. J. Mech. Eng. Autom..

[7] Kamlah M., Böhle U. (2001). Finite element analysis of piezoceramic components taking into account ferroelectric hysteresis behavior. Int. J. Solids Struct..

[8] Delibas B., Arockiarajan A., Seemann W. (2005). A nonlinear model of piezoelectric polcrystalline ceramics under quasi-static electromechanical loading. J. Mater. Sci. Mater. Electron..

[9] Pruvost S., Hajjaji A., Lebrun L., Guyomar D., Boughaleb Y. (2010). Domain switching and energy harvesting capabilities in ferroelectric materials. J. Phys. Chem. C.

[10] Antonio S.Q., Fulginei F.R., Laudani A., Faba A., Cardelli E. (2021). An effective neural network approach to reproduce magnetic hysteresis in electrical steel under arbitrary excitation waveforms. J. Magn. Magn. Mater..

[11] Zhao X., Xie H., Pan H. (2018). Modeling rate-dependent hysteresis in piezoelectric actuators using T-S fuzzy system based on expanded input space method. Sens. Actuators A Phys..

[12] Mohammadzaheri M., Grainger S., Bazghaleh M. (2012). A comparative study on the use of black box modelling for piezoelectric actuators. Int. J. Adv. Manuf. Technol..

[13] Jiles D.C., Atherton D.L. (1986). Theory of ferromagnetic hysteresis. J. Magn. Magn. Mater..

[14] Yu Z., Wu Y., Fang Z., Sun H. (2020). Modeling and compensation of hysteresis in piezoelectric actuators. Heliyon.

[15] Dang X., Tan Y. (2005). Neural networks dynamic hysteresis model for piezoceramic actuator based on hysteresis operator of first-order differential equation. Phys. B Condens. Matter.

[16] Gan J., Zhang X. (2018). An enhanced Bouc-Wen model for characterizing rate-dependent hysteresis of piezoelectric actuators. Rev. Sci. Instrum..

[17] Wang G., Chen G. (2017). Identification of piezoelectric hysteresis by a novel Duhem model based neural network. Sens. Actuators A Phys..

[18] He X., Du H., Tong Z., Wang D., Wang L., Melnik R. (2020). A dynamic hysteresis model based on Landau phenomenological theory of fatigue phenomenon in ferroelectrics. Mater. Today Commun..

[19] Chen Y., Shen X., Li J., Chen J. (2018). Nonlinear hysteresis identification and compensation based on the discrete Preisach model of an aircraft morphing wing device manipulated by an SMA actuator. Chin. J. Aeronaut..

[20] Ma J., Tian L., Li Y., Yang Z., Cui Y., Chu J. (2018). Hysteresis compensation of piezoelectric deformable mirror based on Prandtl–Ishlinskii model. Opt. Commun..

[21] Chen L., Yi Q., Ben T., Zhang Z., Wang Y. (2021). Parameter identification of Preisach model based on velocity-controlled particle swarm optimization method. AIP Adv..

[22] Nam D.N.C., Ahn K.K. (2012). Identification of an ionic polymer metal composite actuator employing Preisach type fuzzy NARX model and Particle Swarm Optimization. Sens. Actuators A Phys..

[23] Galinaitis W., Joseph D., Rogers R. (2001). Parameter identification for Preisach operators with singular measures. Phys. B Condens. Matter.

[24] Hergli K., Marouani H., Zidi M., Fouad Y., Elshazly M. (2019). Identification of Preisach hysteresis model parameters using genetic algorithms. J. King Saud Univ.Sci..

[25] Stakvik J.Å. (2014). Identification, Inversion and Implementaion of the Preisach Hysteresis Model in Nanoposi-Tioning. Master’s Thesis.

[26] Physik Instrumente GmbH, Co. & KG. https://www.physikinstrumente.store/us/p-840.60/.

[27] Physik Instrumente GmbH, Co. & KG. https//www.piusa.us/en/news-events/news/nano-measuring-sensor/.

